# Insight of Silkworm Pupa Oil Regulating Oxidative Stress and Lipid Metabolism in *Caenorhabditis elegans*

**DOI:** 10.3390/foods11244084

**Published:** 2022-12-17

**Authors:** Lina Zhao, Baoxiang Wu, Shuyun Liang, Douyong Min, Hongrui Jiang

**Affiliations:** College of Light Industry and Food Engineering, Guangxi University, Nanning 530004, China

**Keywords:** silkworm pupa oil, antioxidant properties, fat metabolism, *Caenorhabditis elegans*

## Abstract

Silkworm pupa oil (SPO) contains unsaturated fatty acids, tocopherols, and phytosterols, which can regulate serum total cholesterol or be used as an antioxidant. In this study, we investigated the impacts of SPO on the antioxidant stress and lipid metabolism of *Caenorhabditis elegans*. The lifespan of the *C. elegans* fed with different SPO concentrations was determined. The levels of endogenous reactive oxygen species (ROS) were analyzed with the fluorescent probe method. The activity of antioxidant enzymes and the content of malondialdehyde (MDA) were analyzed. The transcription level of specific mRNA was characterized with q-PCR. The survival time of the mutant strain under oxidative stress was determined by *daf-2* (CB1370) mutant, *sod-3* (GA186) mutant, and *skn-1* (EU31) mutant. As for the lipid metabolism, the lipid accumulation was determined with an Oil-Red-O (ORO) staining. The transcription level of specific mRNA was determined by q-PCR. The results showed that the SPO feeding enhanced the activities of antioxidant enzyme by upregulating the expression of the genes skn-1, and sod-3 to decrease the production of ROS and MDA, which prolonged the life of nematodes treated with juglone. ORO staining analysis indicated the feeding of SPO decreased intestinal fat accumulation, downregulated expression of fat-5, fat-6, fat-7, and nhr-80, and upregulated age-1 and tph-1 expression. Conclusively, SPO enhanced the antioxidant capacity by regulating the skn-1 and sod-3 expression of antioxidant gene and reducing the fat accumulation by the insulin/IGF signaling pathway and nuclear hormone receptor nhr-80 signaling pathway of nematodes. This study provides new evidence for the antioxidant and lipid-lowering mechanisms of SPO in *C. elegans*.

## 1. Introduction

Silkworm pupa is a byproduct of the silk reeling industry in China and its outputs are numerous. Insects have been increasingly accepted as a processed ingredient rather than a whole food due to their high-quality protein and fatty acids [1]. A variety of healthcare products made from silkworm pupa have appeared on the market. The dried silkworm pupa contains 30% fat, 75.8% of which is unsaturated fatty acid [2]. Therefore, SPO is considered a new type of edible oil. Some studies have identified essential unsaturated fatty acids from SPO including α-linolenic acid, oleic acid, and linoleic acid [3]. Clinical and animal experiments have shown that unsaturated fatty acids possessing anti-inflammatory, anti-thrombotic, and inhibiting atherosclerosis can reduce cardiovascular mortality, triglyceride level, platelet aggregation, and lower blood pressure, while increasing high-density lipoprotein cholesterol (HDL-C) and improving vascular endothelial function, insulin resistance, and other physiological functions [4,5,6]. The previous studies have confirmed that SPO has antioxidant activity in vitro by scavenging 2,2-diphenyl-1-picrylhydrazine (DPPH) free radicals [7,8]. SPO can reduce serum cholesterol, triglyceride and low-density lipoprotein cholesterol in body, and improve the activity of antioxidant enzymes [9,10]. Polyunsaturated fatty acids in SPO can dose-dependently reduce intracellular total cholesterol and enhance total bile acids, and also increase the conversion of cholesterol to bile acids [11]. However, the previous studies on SPO are mainly focused on the effects of cholesterol metabolism. Insight on the effects of SPO impacting lipid metabolism and antioxidant activity at the molecular level has been rarely reported.

Oxidative stress represents an imbalance between the production of ROS and the biological ability to scavenge ROS. Exogenous oxidants are used to induce a large amount of ROS in *C. elegans*, which makes the nematodes in a sub-dead or dead state; that is, a state known as oxidative stress. The insulin/IGF signaling pathway can regulate fertility, endocrine metabolism, lifespan, and stress response in nematodes [12], where daf-2/daf-16 are the main regulatory genes in the insulin pathway [13]. Skn-1 is a transcription factor homolog encoding mammalian nuclear factor-erythroid-related factor (Nrf), which is regulated by the insulin/IGF signaling pathway. It has been reported that skn-1 plays an important role in ameliorating oxidative stress and regulates downstream genes to prolong lifespan in *C. elegans* [14,15]. The changes in lipid metabolism in the body can cause the accumulation of fat, and many lipids metabolism signaling pathways are conserved in *C. elegans* [16]. For example, the insulin/IGF signaling pathway can regulate the homeostasis of lipid metabolism. The serotonin pathway can control lipid deposition and nematode feeding behavior [17]. The nuclear hormone signaling pathway has the ability to combine fatty acids with some lipids [18].

It has been reported that *C. elegans* can be applied as an ideal model because it is easy to manipulate. For instance, *C. elegans* has been widely used in the fields of genetics, developmental biology, and drug screening due to its small size, short lifecycle, transparent body, specific genetic background, and low maintenance cost. Its genome is completely sequenced, and it has been found that they possess more than 65% genes associated with humans [16]. Technically, juglone, paraquat, and hydrogen peroxide are commonly used as an oxidizing agent. Juglone (5-hydroxyl-1,4-naphthoquinone) is a phenolic compound found in walnuts which can act as a redox cycling agent and produce ROS [19]. When *C. elegans* ingests active substances with antioxidant properties, the endogenous antioxidant defense systems including superoxide dismutase, glutathione peroxidase, glutathione transferase, and catalase have the biological ability to scavenge ROS [20] and resist damage caused by oxidative stress [21,22]. In addition, the glucose diet affects its physiological and molecular processes. It has been reported that the high glucose diet can increase lipid accumulation, increase cellular ROS levels, and shorten lifespan [23]. Due to the transparent nature of the nematode, the accumulation of fat in its intestine can be visualized and quantified by ORO staining [24]. However, the corresponding mechanism has been little reported. Herein, the mechanism of SPO protecting nematodes under oxidative stress induced by juglone was investigated and the route on lipid accumulation of nematodes fed with high glucose diet was elucidated in the current study. Eventually, the SPO regulating oxidative stress and lipid metabolism in *C. elegans* was systematically demonstrated.

## 2. Materials and Methods

### 2.1. Chemicals

ORO was purchased from Beijing Solarbio Science & Technology Co., Ltd. (Beijing, China). Superoxide Dismutase (SOD) assay kit (WST-1 method), catalase (CAT) assay kit, MDA assay kit (TBA method), and Triglyceride (TG) assay kit were purchased from Nanjing Jiancheng Bioengineering Institute (Nanjing, China). BCA Protein Assay Kit was purchased from Beyotime biotechnology (Shanghai, China). α-linolenic acid was purchased from sigma Aldrich Trading Co., Ltd. (Shanghai, China). 5-Hydroxy-1,4-naphthalenedione and 2, 7-dichlorofluorescein diacetate were purchased from Sigma-Aldrich Co. (St. Louis, MO, USA). MiniBEST Universal RNA Extraction Kit, PrimeScript^TM^ RT Master Mix (Perfect Real Time), and TB Green^®^ Premix Ex Taq^TM^ II (Tli RNaseH Plus) were purchased from Takara (Dalian, China). All other chemicals were of analytical grade and used without further purification.

### 2.2. Preparation of SPO

The preparation of SPO has been reported in our previous work [25]. The preparation yield of SPO was of 33%. The main components of SPO were demonstrated in Table 1.

### 2.3. C. elegans Culture and Synchronization

The wild-type (N2) *C. elegans* strain and all mutants of *C. elegans* were obtained from the Caenorhabditis Genetics Center (University of Minnesota, Minneapolis, MN, USA). The mutants were BX107 [*fat-5* (tm420) V], BX106 [*fat-6* (tm331) IV], BX153 [*fat-7* (wa36) V], BX156 [*nhr-80* (tm1011) III], RB1716 [*nhr-49* (hx546)], TJ1052 [*age-1* (hx546)], CB1370 [*daf-2* (e1370) III], GA186 [*sod-3* (tm760) X], EU31 [*skn-1* (zu135) IV/nT1 [unc-?(n754) let-?] (IV; V)]. Unless otherwise stated, *C. elegans* was cultured in nematode growth media (NGM) plates seeded with *E. coli* OP50 at 20 °C. The synchronized *C. elegans* were obtained by lysing gravid adult nematodes with lysis buffer (1 mol NaOH: 7% NaClO: M9 buffer = 10:1:9). After this, the eggs were washed with M9 buffer on NGM plate three times. After 12 h of egg incubation, L1 larvae of the same age were obtained, centrifuged, and transferred to NGM Petri dishes for further cultivation with different conditions for subsequent experiments.

For each assay, the plates were prepared as follows [26].

The control plate: the solution prepared with OP50 and DMSO-M9 (4:1, *v*/*v*) was spread on a NGM plate.

The SPO plate: the solution prepared with OP50 and different concentrations of SPO solution (4:1, *v*/*v*) was spread on the NGM plates to prepare the SPO plates with 0.05, 0.1, and 0.5 mg/mL concentration.

As for the fat accumulation assay, the different NGM plates containing 10 mM glucose were prepared as follows.

The high-fat model plate: the solution prepared with OP50 and DMSO-M9 (4:1, *v*/*v*) was spread on the NGM plate containing glucose.

The high-fat and SPO plate: the solution prepared with OP50 and different concentrations of SPO (4:1, *v*/*v*) was spread on the NGM plate containing glucose to prepare the SPO plates with 0.1 and 0.5 mg/mL concentration.

### 2.4. Life Span Assay

The N2 nematodes at L1 stage were inoculated in the NGM plate which contained 50 μM 5-FUdR to prevent offspring generation. After 44 h, the nematodes grew to L4 stage, and the culture plate was changed daily. As a comparison, the nematodes were inoculated in the NGM plates containing *E. coil* OP50 with different concentrations or without SPO. The inoculation was maintained at 20 °C, and the survival status was examined daily with a stereomicroscope (LEICA Microsystems CMS Gmbh, Wetzlar, Germany) until all nematodes died. Thirty nematodes were used for every experiment, of which there were three replications.

### 2.5. Juglone Resistance Assay

The oxidative stress of *C. elegans* was determined according to the reported protocol [27] with minor modifications. Briefly, the N2 and the mutant strain nematodes (CB1370 [daf-2(e1370) III], GA186 [sod-3(tm760) X], EU31 [skn-1(zu135) IV/nT1 [unc-?(n754) let-?] (IV; V)]) at L4 stage were inoculated in the NGM plate with or without SPO for 24 h. After that, the corresponding nematodes were transferred to the NGM plate with 200 μM juglone, and the living nematodes were counted every one hour by a stereomicroscope. The cultivation temperature was 20 °C. The experiment was replicated three times.

### 2.6. Endogenous ROS Assay

The accumulation of ROS in nematodes was determined according to the reported protocol [28]. Briefly, the L4 stage nematodes cultivated with different conditions were completely washed with M9 buffer, and then the nematodes were suspended in 1 mL M9 buffer. Fifty μL of the suspension was transferred to the black 96-well plate, and then 50 μL of 100 μM H_2_DCF-DA solution was added. The nematode suspension without H_2_DCF-DA was applied as the control. The fluorescence intensity was recorded every 30 min within 1.5 h with a microplate reader (Infinite^®^ M200 pro, TECAN, Männedorf, Switzerland) at 20 °C (the excitation wavelength was 485 nm and the emission wavelength was 530 nm). As the nematode was lysed, the protein concentration was determined by a BCA kit. The level of ROS was defined by fluorescence/protein.

### 2.7. Fat Accumulation Analysis

The fat accumulation of *C. elegans* was detected by ORO staining according to the described protocol [28]. The L4 stage nematodes cultivated with different conditions were completely washed with M9 buffer. The ORO stock solution was diluted with deionized water (ORO stock solution: deionized water = 6:4, *v*/*v*) and filtrated by 0.45 μM filter to remove impurities. The nematodes were washed with M9 buffer, and then centrifuged at 1000 G for 2 min to remove water. After that, 500 μL of 60% isopropanol was used to dehydrate and fix the nematodes. Subsequently, the centrifugation was applied to obtain the nematodes which was stained with 500 μL of ORO solution for 12 h. The stained nematodes were washed with M9 buffer and then photographed with a microscopy (DMi8 manual, LEICA, Wetzlar, Germany). Fifteen nematodes were photographed in each experimental group. Images were analyzed using the Image J software.

### 2.8. Quantification of T-SOD, CAT, MDA and TG

After the excessive *E. coli* OP50 was completely washed with M9 buffer, the nematodes were transferred and crushed with a glass homogenizer within a tube in an ice bath. The suspension was centrifuged according to the instructions of the kit, and the supernatant was extracted to measure TG, T-SOD, CAT, and MDA. The protein concentration was determined with the BCA kit to standardize the activity of antioxidant enzymes and TG levels.

### 2.9. Gene Expression

The RNA was extracted from the nematodes as follows. Briefly, as the nematodes were washed with M9 buffer, the total RNA samples of nematodes were extracted with the Takara minibest universal RNA extraction kit according to the instruction. Subsequently, the total RNA of nematodes was reverse transcribed into cDNA using primescript^TM^ RT Master Mix (perfect real time) of Takara. TB green was used for real-time fluorescence quantitative PCR analysis. *Premix Ex Taq*^TM^ II (TLI RNaseH plus) was performed on Lightcycler96 analysis system. The primer sequences of all genes and β-actin used as an internal control were listed in Table 2. The relative quantification of genes was determined using the 2^−ΔΔCt^ method. The experiment was replicated three times.

### 2.10. Statistical Analysis

All experiment tests were replicated three times, and SPSS 25.0 was used to perform statistical analysis. The lifespan curves were estimated using Kaplan-Meier method with GraphPad Prism 9. The results were presented as the mean ± SEM for the group with large number of samples (*n* ≥ 10) or as the mean ± SD for the group with small number of samples. The significance of the difference between the two groups was verified with the Student’s *t*-test. ANOVA was used for comparisons multiple groups. As all comparisons were made to 1 control group, the Dunnett’s method was used for post-hoc. As all comparison groups were compared in pairs, the Tukey’s method was used for post-hoc. Significance was established at *p* < 0.05.

## 3. Results and Discussion

### 3.1. The Effect of SPO on C. elegans Lifespan and Oxidative Stress

**Figure 1:**
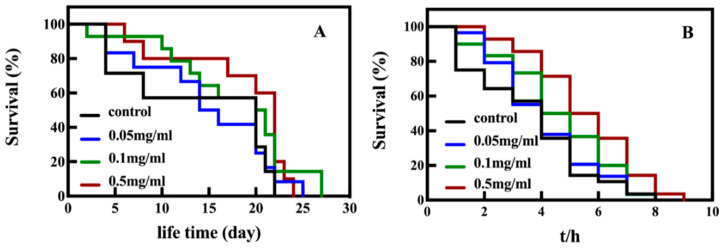
Impact of SPO on lifespan of *C. elegans* (**A**). Impact of SPO on lifespan of *C. elegans* treated with 200 μM juglone (**B**).

The wild-type *C. elegans* N2 was applied for evaluating the antiaging impact of SPO. Figure 1A shows the SPO feeding can prolong the lifespan of nematodes. Table 3 shows that the nematodes cultivated with 0.1 mg/mL SPO exhibited the longest lifespan (the average lifespan was 18.87 days, the maximum lifespan was 25 days), which can be attributed to the bioactive components of SPO. It has been reported that linolenic acid and oleic acid were the main bioactive components of SPO regulating the metabolism of nematodes [11,29].

Juglone as a redox quinone can generate ROS inducing severe oxidative damage [30]. The survival of nematodes under oxidative stress was shown in Figure 1B and Table 4. It was observed that the SPO feeding improved the survival rate of nematodes under the juglone-induced oxidative damage which can be attributed to the radical scavenger of SPO. The in vitro antioxidant assay revealed that DPPH IC50 and ABTS IC50 of SPO were 37.63 mg/mL and 92.42 mg/mL, respectively [25]. The result indicated that the nematodes fed with 0.5 mg/mL SPO exhibited the survival time which was of 5.51 h (*p* < 0.05). Conclusively, the application of SPO can significantly improve the lifespan and attenuate the oxidative damage of nematodes.

### 3.2. Effect of SPO on Endogenous Oxidative Stress and Antioxidant Enzymes

**Figure 2:**
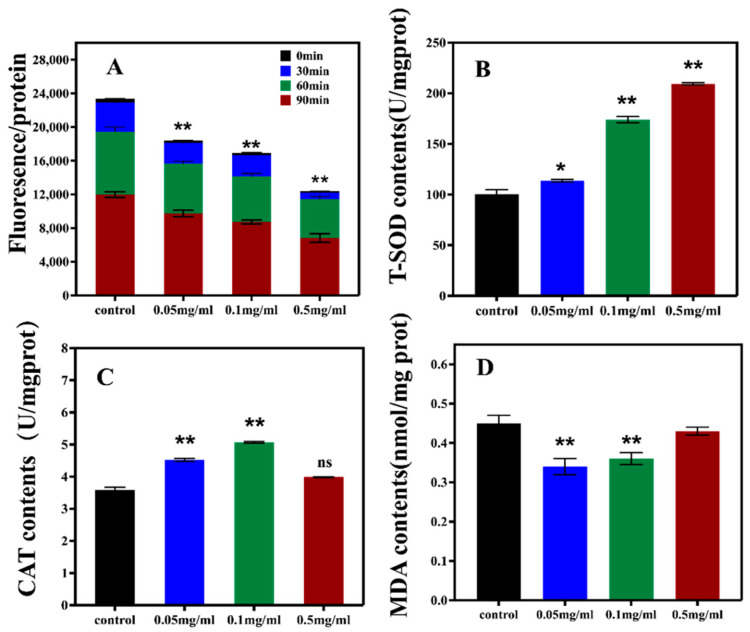
Effect of SPO on ROS (**A**), activity of SOD (**B**) and CAT (**C**), and MDA contents (**D**). All data are expressed as mean ± SD, analyzed with ANOVA with the Dunnett’s comparisons test (post-hoc), relative to control * *p* < 0.05, ** *p* < 0.01, ns—no significance.

The impacts of SPO on the endogenous ROS production, antioxidant enzyme activity, and lipid peroxide products of nematodes were evaluated to further elucidate the antioxidant mechanism of SPO. 2′,7′-dichlorodihydro fluorescein diacetate (H2DCF-DA) was applied to quantify the endogenous ROS levels in nematodes. Compared to the control, the ROS fluorescence intensity of nematodes decreased with the increasing SPO feeding within 0.05–0.5 mg/mL (Figure 2A), indicating that SPO can reduce the endogenous ROS production.

The impacts of SPO on the activities of SOD & CAT and the content of MDA were demonstrated in Figure 2B–D. Compared with the control, the activity of SOD of nematodes fed with different concentrations SPO increased by 13.51% (*p* < 0.05), 73.52% (*p* < 0.05), and 108.82% (*p* > 0.05), respectively. The activity of CAT increased by 26.52%, 46.61%, and 11.43% (*p* < 0.05), respectively. Meanwhile, the content of MDA decreased by 23.65%, 20.91% (*p* < 0.05), and 3.42% (*p* > 0.05), respectively. It has been reported that oleic acid (OA) can extend the life of *C. elegans* by significantly increasing the activities of superoxide dismutase, catalase, and glutathione peroxidase. Therefore, it was speculated that SPO containing oleic acid also can enhance the activity of antioxidant enzymes to prolong the life of nematodes. The results suggested that the SPO feeding can attenuate the damage of ROS by increasing the activity of antioxidant enzymes in nematodes.

### 3.3. Effect of SPO on Oxidative Stress-Related Gene Expression

**Figure 3:**
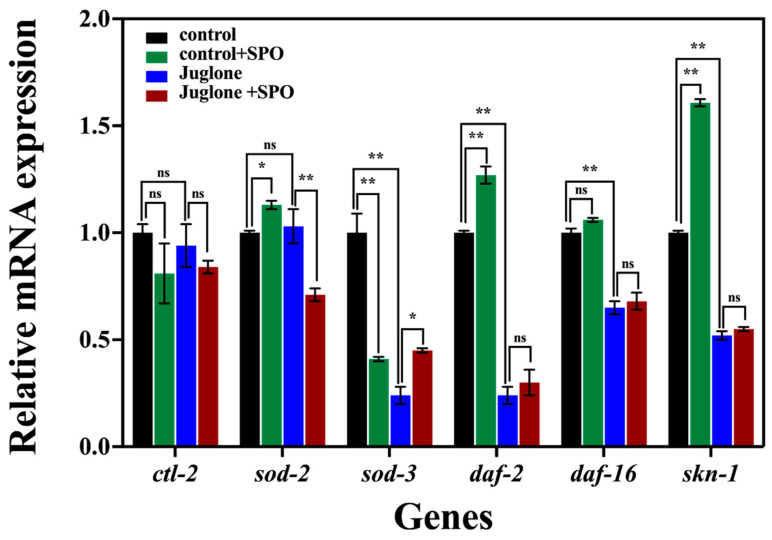
Effect of SPO on expression of antioxidant genes. All data are expressed as mean ± SD, analyzed with ANOVA with the Tukey’s comparisons test (post-hoc), relative to control * *p* < 0.05, ** *p* < 0.01.

The expression level of SPO on the antioxidant genes of nematodes were evaluated to further elucidate the antioxidant mechanism of SPO (Figure 3).

Compared with the control, the expression level of sod-2, daf-2, and skn-1 genes of nematodes fed with SPO was upregulated by 1.13, 1.27, and 1.61 times, respectively (*p* < 0.05). The expression level of sod-3 gene was downregulated by 0.41 times (*p* < 0.05), and the expression level of daf-16 and ctl-2 genes was little changed (*p* > 0.05). Skn-1 is the key gene for oxidative stress resistance which regulate the transcription of NADH quinone oxidoreductase, the CAT ctl-1, and the SOD (sod-1, sod-2, and sod-3) [31]. It has been reported that the oxide of linolenic acid and oleic acid can enhance the transcription of skn-1 gene and the transcription of sod-2 gene, respectively [29,32]. The results suggested that skn-1 and sod-2 are the key genes for the anti-aging effect of SPO.

Compared with the control, the expression level of sod-3, daf-16, daf-2, and skn-1 genes of nematodes fed with juglone were down-regulated by 0.52, 0.65, 0.24, and 0.24 times, respectively (*p* < 0.05), while the expressions level of sod-2 and ctl-2 were not significantly changed (*p* > 0.05). Juglone as an arylating agent can inhibit the transcription of daf-16 and skn-1 [33]. The results suggested that the transcription of skn-1, sod-3, daf-16, and daf-2 genes were inhibited with juglone.

Compared to the sample treated with juglone, the expression level of sod-3 gene of nematodes treated with juglone and SPO was up-regulated by 1.10 times (*p* < 0.05), the expression level of daf-2, daf-16, and skn-1 and ctl-2 genes were not significantly changed (*p* > 0.05). The expression level of sod-2 gene was down-regulated by 0.69 times (*p* < 0.05). The result suggested that sod-3 is the key gene for the anti-oxidative stress effect of SPO. The results suggested that SPO activated sod-3 gene to resist the oxidative stress damage induced with juglone.

### 3.4. Effect of SPO on Oxidative Stress of Mutants Induced with Juglone

**Figure 4:**
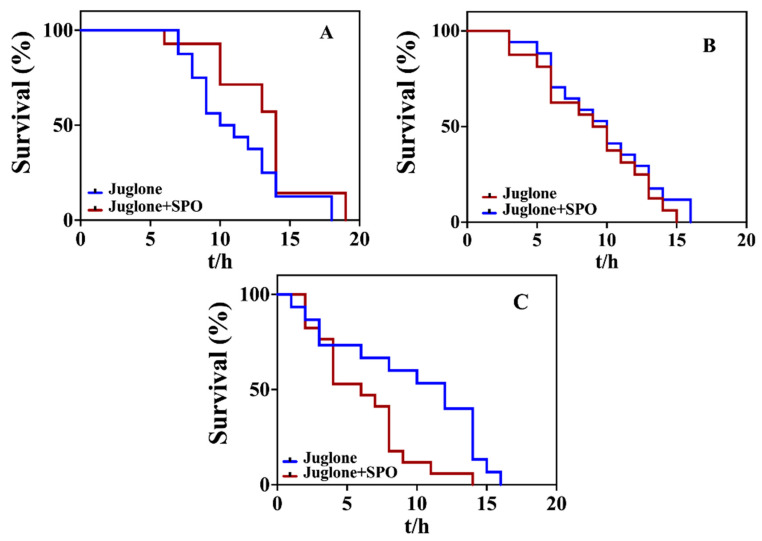
Effect of SPO on the oxidative stress of *daf-2* (CB1370) mutant (**A**), *sod-3* (GA186) mutant (**B**) and *skn-1* (EU31) mutant (**C**) induced with juglone.

*Daf-2* (CB1370) mutant, *skn-1* (EU31) mutant and *sod-3* (GA186) mutant were used to investigate the effects of survival time of *C. elegans* under the oxidative stress induced with juglone (Figure 4). Table 5 shows that the mean lifespan of *daf-2* (CB1370) mutant with SPO feeding were increased by 316.00% (*p* < 0.05) and the maximum lifespan were extended by 1 h compared to the sample treated with juglone (Figure 4A). The mean lifespan of *skn-1* (EU31) mutant and *sod-3* (GA186) mutant with SPO feeding was reduced by 45.10% (*p* < 0.05) and 95.79% (*p* > 0.05), and the maximum lifespan were reduced by 2 h and 1 h (Figure 4B,C). It was demonstrated that under the oxidative stress induced with juglone, the SPO feeding can prolong the lifespan when daf-2 gene was deleted, while hardly prolong the lifespan as skn-1 and daf-2 genes were deleted. The results revealed that sod-3 and skn-1 were key genes for SPO attenuating the oxidative stress induced with juglone.

### 3.5. Effects of SPO on Fat and TG Accumulation

**Figure 5:**
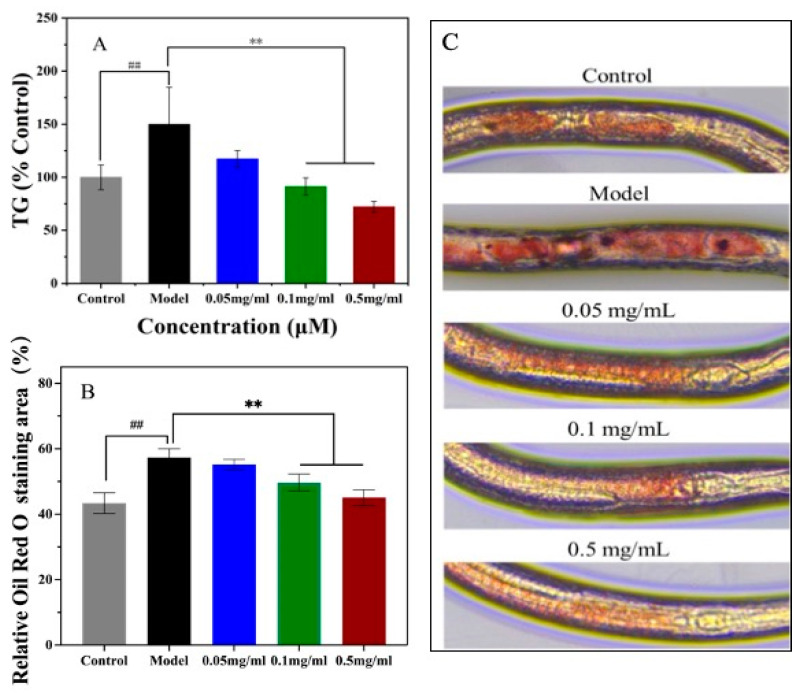
Effect of SPO on triglycerides (**A**), ORO staining of *C. elegans* (**B**) Effects of SPO on fat accumulation (**C**). All data are expressed as mean ± SD, analyzed with ANOVA with the Dunnett’s comparison test (post-hoc), relative to control ** *p* < 0.01; relative to model ## *p* < 0.01.

The impact of SPO feeding on triglycerides (TG) levels in nematodes was demonstrated in Figure 5A. It was observed that the in vivo TG levels of nematodes were decreased with the increasing SPO feeding. For example, compared with the model, the TG level decreased by 21.73%, 39.07%, and 51.85% (*p* < 0.05) as 0.05, 0.1, and 0.5 mg/mL SPO were fed accordingly. The result indicated that the SPO feeding reduced the TG levels of nematodes.

The fat accumulation in nematodes characterized by the ORO staining was demonstrated in Figure 5B,C. It was observed that the area of lipid droplets in the model was higher than that of the control (*p* < 0.05). The comparable results have been reported that the lipid droplets stored in intestinal and subcutaneous cells of *C. elegans* were clearly observed with ORO staining [34,35]. It was observed that the color of nematodes with SPO feeding was lighter than that of the model group, and the color decreased with the increasing SPO feeding. The result confirmed that SPO can attenuate the fat accumulation in *C. elegans*.

### 3.6. Effects of SPO on Regulating Fat Metabolism

**Figure 6:**
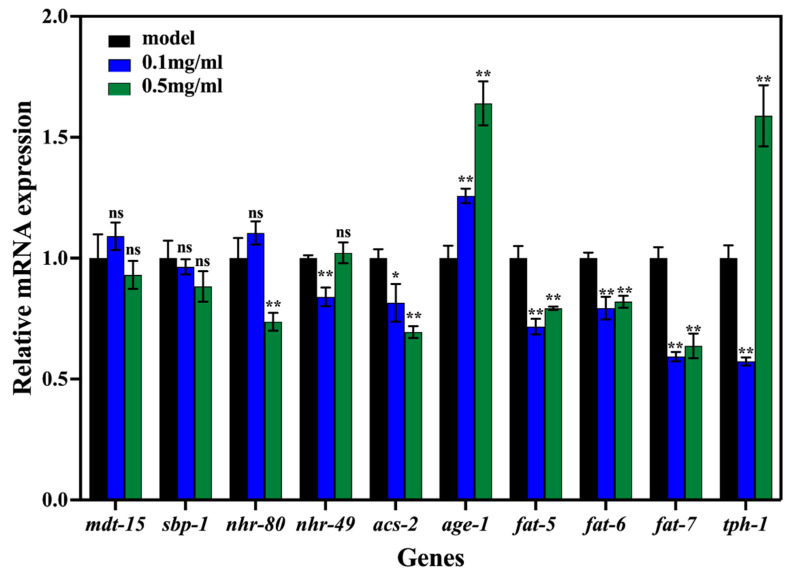
Effects of SPO on the expression of lipid metabolism genes. All data are expressed as mean ± SD, analyzed with ANOVA with the Dunnett’s comparisons test (post-hoc), relative to control * *p* < 0.05, ** *p* < 0.01, ns—no significance.

The expression level of fat metabolism-related genes of *C. elegans* affected by the SPO feeding was demonstrated in Figure 6. Compared with the model, the expression levels of fat-5, fat-6, fat-7, and acs-2 genes were down-regulated as nematodes were fed with SPO (*p* < 0.05). Three delta 9-desaturase were identified in *C. elegans*. The fat-5 gene is a palmitic acid desaturase converting palmitic acid to palmioleic acid. The fat-6 and fat-7 genes are the stearic acid desaturases converting stearic acid to oleic acid. It has been reported that the transcription of fat-6 contributes to the production of OA. Palmitoleic acid and oleic acid are the essential substrates for the synthesis of triglycerides. Therefore, when the expression level of 9-desaturase factors was upregulated, this promoted the accumulation of fat in nematodes [36,37]. Acs-2 is an acyl CoA synthase that can catalytically convert fatty acids to acyl CoA β [36,38,39]. In this study, the acs-2 gene expression level decreased with the increasing SPO feeding. It was speculated that the decrease of fat content attenuated the transcription response reducing the expression level of acs-2 which in turn limited further energy consumption [38].

Compared with the model, the expression level of age-1 gene was upregulated with the SPO feeding (*p* < 0.05). Age-1 is a homologue of the mammalian phosphatidylinositol 3-OH kinase catalytic subunit (PI3K), which activates serine/threonine kinase 1(AKT-1) to regulate metabolic enzyme activity and glucose transport. The absence of age-1 attributed to the accumulation of fat in *C. elegans* [40].

Compared with the model, the expression level of nhr-49 gene was not significantly changed with the SPO feeding (*p* > 0.05), but the expression level of nhr-80 gene was down-regulated by 0.74 times as 0.5 mg/mL SPO was fed (*p* < 0.05). Nhr-80 and nhr-49 are the nuclear hormone receptors in *C. elegans* which regulate the delta 9-desaturase transcription [35,36,40]. It has been reported that the absence of fat-6 or fat-7, nhr-80 can up-regulate the fat-5 gene transcription [37].

Compared with the model, the expression level of the tph-1 gene was down-regulated by 0.57 times as 0.1 mg/mL SPO was fed (*p* < 0.05), but the expression level of tph-1 was up-regulated by 1.59 times as 0.5 mg/mL SPO was applied (*p* < 0.05). Tph-1 is a critical gene for the biosynthesis of 5-hydroxytryptophanase (5-HT), which inhibits dietary intake and reduces body weight of *C. elegans* [35,41].

Compared with the model, the expression levels of mdt-15 and sbp-1 genes were not significantly changed with the SPO feeding (*p* < 0.05). Mdt-15 functions as a co-regulator of transcription factors regulates lipid metabolism and stress resistance [42]. It has been reported that the sbp-1/mdt-15 signaling pathway regulates the transcription of delta-9 desaturase and attenuates the transcription of fat-6 and fat-7 genes [43]. As a result, it was revealed that the SPO feeding can regulate the transcription of genes including fat-5, fat-6, fat-7, age-1, acs-1, nhr-80, and tph-1.

### 3.7. Effect of SPO on TG Level in Mutants

**Figure 7:**
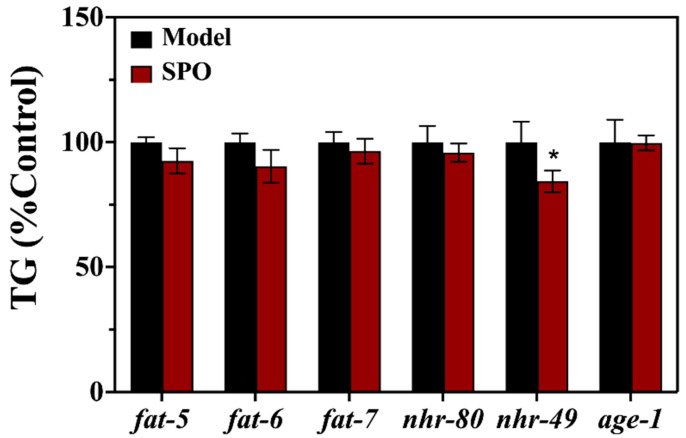
Effects of SPO on TG in mutants. All data are expressed as mean ± SD. The Student’s *t*-test was used for comparison, relative to model * *p* < 0.05.

The mutant strains of fat-5, fat-6, fat-7, nhr-80, and age-1 were used to investigate the effects of SPO feeding on TG levels. Figure 7 shows that the TG level of *fat-5* (BX107) mutant, *fat-6* (BX106) mutant, *fat-7* (BX153) mutant, *nhr-80* (RB1716) mutant and *age-1* (TJ1052) mutant was not significantly changed as the nematodes was fed with SPO compared to the models (*p* > 0.05). However, the TG level of *nhr-49* (BX165) mutant was significantly decreased (*p* < 0.05). The SPO feeding little reduced fat accumulation as fat-5, fat-6, fat-7, nhr-80, and age-1 genes were deleted. However, the SPO feeding can reduce fat accumulation as nhr-49 gene was deleted. The results suggested that the SPO feeding reduced the fat accumulation of *C. elegans* via the fat-5, fat-6, fat-7, nhr-80, and age-1 signaling.

## 4. Conclusions

The SPO feeding increased the activities of antioxidant enzymes SOD and CAT, reduced the production of ROS and MDA, and improved the anti-stress ability through the skn-1/sod-3 signaling pathway. The SPO feeding also promoted glucose transfer and fatty acid desaturation and reduced intestinal lipids accumulation through insulin signaling pathway and nuclear hormone receptor nhr-80 signaling pathway. The current investigation provides the theoretical knowledge for the application of SPO as a nutritional additive and even as an antioxidant or lipid-lowering drug.

## Figures and Tables

**Table 1:** The composition of SPO.

Polyunsaturated Fatty Acids	Relative Content %
Palmitic acid	31.53 ± 1.20
Stearic acid	15.31 ± 0.78
Oleic acid	14.79 ± 0.51
Linoleic acid	5.59 ± 0.22
n3 α-linolenic acid	30.46 ± 1.18

**Table 2:** Sequences of primers used in the gene expression analysis.

Gene	Forward (5′-3′)	Reverse (5′-3′)
*act-1*	ACGAGGTTGCCGCTCTTGTTG	ACGAGTCCTTCTGTCCCATACCG
*ctl-2*	TGAGGTTGAACAATCCGCCTTCTG	CGTCCTTGGAGCATCTTGTCTGG
*sod-2*	GCTCTTCAGCCAGCTCTCAAGTTC	ACTCCGCCGATGGTTCTCCTC
*sod-3*	AGCATCATGCCACCTACGTGA	CACCACCATTGAATTTCAGCG
*daf-2*	CCACGACGACGAGCACATCAC	GGCGGGTTTTCCTCATAGCAGTC
*daf-16*	ATCATGGGTTGGCGAATCGGTTC	CGGCTTCGACTCCTGCTTAATCTG
*skn-1*	GGTCTCCGTTGGCGTGATGATC	CTGGTGGATGCTCGGTGAGTATTG
*fat-5*	CGGCCGCCCTCTTCCGTTAC	TGGCTGCCATCCGACCCAGT
*fat-6*	TCAACAGCGCTGCTCACTAT	TTCGACTGGGGTAATTGAGG
*fat-7*	CAACAGCGCTGCTCACTATT	CACCAACGGCTACAACTGTG
*nhr-49*	AGGCTCGTGTCAATCAAGAGATGTG	ATGCCGATGCTCCAGAATCACTTC
*acs-2*	GCAGCCTCGCTCTACACTCT	GACTCCTGCAAATGCACATGC
*mdt-15*	GAGCACCACTACCAGGTGGT	ACCAGCAATGGGTCAGCCAC
*sbp-1*	AATCTGGGTTTGGCGGTTGGC	CGAGCGACTTCTTTGTGTGAATGC
*age-1*	GCTGCTCCGTGCAGAGATTG	CACGGAGGTAAGCTTCCATC
*nhr-80*	TGAGGTTCAGGAGCCAAATAG	GAAGGAGGTGGACGATGAGA
*tph-1*	CGGTGAGCCAATTCCGCGAA	AGAAACTGCTTGCATGCGTGC

Note: Actin was used as the control.

**Table 3:** Impact of SPO on the lifespan of *C. elegans*.

Groups	Mean Lifespan (Day)	Maximum Lifespan (Day)
control	15.50 ± 0.24	23.5 ± 1.00
0.05 mg/mL	16.73 ± 0.09 *	24.9 ± 0.82
0.1 mg/mL	18.78 ± 0.09 *	25.5 ± 1.5
0.5 mg/mL	18.40 ± 0.15 *	24.0 ± 2.16

Note: All data are expressed as mean ± SEM, analyzed with ANOVA with the Dunnett’s test (post-hoc), relative to control * p < 0.05 (*n* = 30).

**Table 4:** Impact of SPO on the antioxidative damage in *C. elegans*.

Groups	Mean Lifespan (h)	% of Control
control	3.8 ± 0.40	-
0.05 mg/mL	4.15 ± 0.23 ^ns^	109.21%
0.1 mg/mL	4.49 ± 0.25 *	118.16%
0.5 mg/mL	5.51 ± 0.02 *	145.00%

Note: All data are expressed as mean ± SEM, analyzed with ANOVA with the Dunnett’s comparisons test (post-hoc), relative to control * *p* < 0.05 (*n* = 25), ^ns^ stands for not significant.

**Table 5:** Effect of SPO on the oxidative stress of mutant strain induced with juglone.

Mutant Strain	Groups	Mean Lifespan (h)	% of Control
*daf-2* (CB1370)	Juglone	5.13 ± 0.24	-
	Juglone + SPO	8.29 ± 0.03 *	316.00%
*sod-3* (GA186)	Juglone	6.90 ± 0.18	-
	Juglone + SPO	6.61 ± 0.22	95.79%
*skn-1* (EU31)	Juglone	7.76 ± 0.23	-
	Juglone + SPO	3.50 ± 0.01 *	45.10%

Note: All data are expressed as mean ± SEM. The Student’s *t*-test was used for comparison, relative to control * *p* < 0.05 (*n* = 25).

## Data Availability

The data presented in this study are available on request from the corresponding author.
